# Phosphorylation of Drebrin by Cyclin-Dependent Kinase 5 and Its Role in Neuronal Migration

**DOI:** 10.1371/journal.pone.0092291

**Published:** 2014-03-17

**Authors:** Kazuya Tanabe, Hiroyuki Yamazaki, Yutaka Inaguma, Akiko Asada, Taeko Kimura, Junya Takahashi, Masato Taoka, Toshio Ohshima, Teiichi Furuichi, Toshiaki Isobe, Koh-ichi Nagata, Tomoaki Shirao, Shin-ichi Hisanaga

**Affiliations:** 1 Department of Biological Sciences, Tokyo Metropolitan University, Hachioji, Tokyo, Japan; 2 Department of Neurobiology and Behavior, Gunma University Graduate School of Medicine, Maebashi, Gunma, Japan; 3 Molecular Neurobiology, Institute for Developmental Research, Kasugai, Aichi, Japan; 4 Department of Chemistry, Tokyo Metropolitan University, Hachioji, Tokyo, Japan; 5 Department of Life Science and Medical Bio-Science, Waseda University, Tokyo, Japan; 6 Department of Applied Biological Science, Faculty of Science and Technology, Tokyo University of Science, Noda, Chiba, Japan; Osaka University Graduate School of Medicine, Japan

## Abstract

Cyclin-dependent kinase 5 (Cdk5)-p35 is a proline-directed Ser/Thr kinase which plays a key role in neuronal migration, neurite outgrowth, and spine formation during brain development. Dynamic remodeling of cytoskeletons is required for all of these processes. Cdk5-p35 phosphorylates many cytoskeletal proteins, but it is not fully understood how Cdk5-p35 regulates cytoskeletal reorganization associated with neuronal migration. Since actin filaments are critical for the neuronal movement and process formation, we aimed to find Cdk5 substrates among actin-binding proteins. In this study, we isolated actin gels from mouse brain extracts, which contain many actin-binding proteins, and phosphorylated them by Cdk5-p35 *in vitro*. Drebrin, a side binding protein of actin filaments and well known for spine formation, was identified as a phosphorylated protein. Drebrin has two isoforms, an embryonic form drebrin E and an adult type long isoform drebrin A. Ser142 was identified as a common phosphorylation site to drebrin E and A and Ser342 as a drebrin A-specific site. Phosphorylated drebrin is localized at the distal area of total drebrin in the growth cone of cultured primary neurons. By expressing nonphosphorylatable or phosphorylation mimicking mutants in developing neurons *in utero*, the reversible phosphorylation/dephosphorylation reaction of drebrin was shown to be involved in radial migration of cortical neurons. These results suggest that Cdk5-p35 regulates neuronal migration through phosphorylation of drebrin in growth cone processes.

## Introduction

Mammalian neurons display active cellular movements and dynamic morphological changes during brain development [1], [2]. These movements consist of neuronal migration from their birthplace to resident position, outgrowth of axon and dendrites, and spine formation, all of which are critical for establishment of proper neuronal network formation. Dynamic reorganization of cytoskeletons, both microtubules and actin filaments, is required for these movements and morphological changes [3], [4]. In addition to the distinct regulation of each cytoskeleton, harmonized control of two cytoskeletal systems should be maintained in complex modes of neuronal migration; however, both distinct and coordinated regulations of cytoskeletons are insufficiently understood in terms of migrating neurons.

Cyclin-dependent kinase 5 (Cdk5) is a proline-directed Ser/Thr kinase that is activated by the neuron-specific regulatory subunits p35 and p39 [5]–[7]. Cdk5-deficient mice show abnormal lamination in the brain with perinatal lethality [8]. Mice lacking p35 also show disrupted neuronal layers in the cerebral cortex, although they can survive [9]. Thus, Cdk5 activity is critical for proper brain development. Cdk5-p35 phosphorylates many cytoskeletal proteins related to both microtubules and actin filaments [10]–[14], but it is thought that Cdk5 more directly regulates microtubules; less is known about how Cdk5 regulates actin filaments. Actin filaments function mainly in the subplasma membrane region. Considering the localization of active Cdk5-p35 on membranes, it is highly likely that actin filaments are a major target for Cdk5; if so, it is important to identify target proteins in actin cytoskeletons for Cdk5. Although a number of actin-binding proteins have been shown to be phosphorylated by Cdk5 [12]–[14], a few have been shown to be involved in neuronal development. Identifying Cdk5 substrates from actin cytoskeletal proteins remains unexplored.

Drebrin is a side-binding protein of actin filaments [15], which competes with the several F-actin binding proteins such as α-actinin, tropomyosin, fascin, and myosin [16]. There are two isoforms of drebrin: a ubiquitously expressed isoform drebrin E and a neuron-specific isoform drebrin A. Drebrins are expressed in the developing brain and are involved in neurite outgrowth and spine formation through the remodeling of actin filaments [17]–[22]. *In vitro* characterization of drebrin has been previously described but its regulation has yet to be investigated. Phosphorylation of drebrin is indicated in non-neural cells [23], [24]. Although neuronal phosphorylation is also suggested [25], the exact phosphorylation site(s), its kinase, and role remain unknown.

In this study, we found that drebrin is phosphorylated by Cdk5-p35. We additionally identified Ser142 and Ser342 in drebrin at Cdk5-phosphorylation sites and identified the role of neuronal migration in the embryonic cortex.

## Materials and Methods

### Ethics Statement

All animal experiments were performed according to the guidelines for animal experimentation of Tokyo Metropolitan University. The study was approved by the Research Ethics Committee of Tokyo Metropolitan University (approval number, 24–45). All efforts were made to reduce the suffering of animals used.

### Antibodies and Chemicals

Monoclonal anti-drebrin (M2F6) and monoclonal anti-GFP were purchased from MBL (Nagoya, Japan). Monoclonal anti-neuron-specific class III β-tubulin (Tuj1) was obtained from Genzyme-Techne (Minneapolis, MN), anti-actin was obtained from Sigma-Aldrich (St Louis, MO), and monoclonal anti-myc (4A6) was obtained from Millipore (Billerica, MA). Anti-Homer2a was used as described previously [26]. Horseradish Peroxidase (HRP)-conjugated anti-mouse IgG and HRP-conjugated anti-rabbit IgG were purchased from DAKO (Glostrup, Denmark). Alexa Fluor 488 goat anti-mouse IgG, Alexa Fluor 488 goat anti-rabbit, Alexa Fluor 546 goat anti-rabbit IgG, Alexa Fluor 647 goat anti-mouse, and Alexa Fluor 647 goat anti-rabbit were purchased from Invitrogen (Carlsbad, CA). Tetramethylrhodamine isothiocyanate-phallidin (TRITC-phalloidin) was purchased from Sigma-Aldrich. And roscovitine and Phos-tag acrylamide were obtained from Wako (Osaka, Japan).

### Production and Purification of Anti-phospho-Ser142 of Drebrin

Phospho-Ser142 peptide C-LARLS(pS)PVHR and non-phospho-Ser142 peptide C-LARLSSPVHR were obtained from BEX (Tokyo, Japan). The keyhole limpet hemocyanin (KLH)-conjugated phospho-Ser142 peptide was immunized into 15-weeks-old female rabbits (New Zealand White, Sankyo Labo, Tokyo, Japan) using TiterMax Gold (Funakoshi, Tokyo, Japan) as adjuvant. The anti-phospho-Ser142 antibody (pS142) was purified from antiserum using a phospho-Ser142-bound column after removing non-phospho-antibody with a Ser142 peptide-bound column.

### Plasmids Construction

HA-Cdk5, kinase-negative HA-Cdk5 (Cdk5-D144N), and p35-myc have been described previously [27], [28]. pEGFP-drebrin A, pEGFP-drebrin E, and pEGFP-ins2 have also been described previously [25]. The N-terminal fragment (pEGFP-drebrin A-NT) and C-terminal fragment (pEGFP-drebrin-CT) of Drebrin A were constructed by PCR-based deletion mutagenesis using pEGFP-drebrin A as a template. The primers used were:

5′-GATCTCGAGGCATGGCCGGCGTCAGC-3′ and 5′-TTGGATCCCTACAGTCTGGGAGCAGACTCC-3′ as forward and reverse primers, respectively, for the N-terminal fragment, 5′-TAACTCGAGGCATGGACGGTGAAGAGGTCTGC-3′ and 5′-GTGGATCCCTAATCACCACCCTCGAA-3′ for the C-terminal fragment. Nonphosphorylation Ala and phosphorylation-mimic Asp mutants of drebrins were constructed by site-directed mutagenesis using pEGFP-drebrin A, pEGFP-drebrin E, pEGFP-drebrin A-NT, pEGFP-drebrin-CT, pEGFP-ins2 and pET19b-drebrin A and pET19b-drebrin E as templates. The primers used were as follows:

5′-TCGGCTCTCTGCCCCAGTGCTGC-3′ and 5′-GCAGCACTGGGGCAGAGAGCCGA-3′ for S142A, 5′-CTTCCTCCTCTGCCCCTCCACG-3′ and 5′-CGTGGAGGGGCAGAGGAGGAAG for S342A, 5′-CCTCCACGGGCTCCCTTTCCC-3′ and 5′-GGGAAAGGGAGCCCGTGGAGG-3′ for T346A, 5′-CCTGCCACCGCGCCCCAAACCT-3′ and 5′-AGGTTTGGGGCGCGGTGGCAGG-3′ for T356A, 5′-ATGGCGCCCGCTCCCATTCCCA-3′ and 5′-TGGGAATGGGAGCGGGCGCCAT-3′ for T377A, 5′-ATTCCCACCCGGGCCCCATCTGATT-3′ and 5′-AATCAGATGGGGCCCGGGTGGGAAT-3′ for S383A, 5′-ACAGCCTCCGCCCCCATCACGGA-3′ and 5′-TCCGTGATGGGGGCGGAGGCTGT-3′ for T392A, 5′-TCGGCTCTCTGACCCAGTGCTGC-3′ and 5′-GCAGCACTGGGTCAGAGAGCCGA-3′ for S142D, 5′-CTTCCTCCTCTGACCCTCCACG-3′ and 5′-CGTGGAGGGTCAGAGGAGGAAG-3′ for S342D. pEGFP-drebrin A-WT, S142A/S342A, and S142D/S342D were subcloned into *BamHI* site of pCAG-GFP-MCS2 [29], and DsRed-monomer derived from pDsRed-monomer-N1 was subcloned into *XhoI* site of pCAGGS. Mutations were confirmed by DNA sequences.

### Expression and Purification of Recombinant Drebrin

pET19b-drebrin A, pET19b-drebrin E, and their Ala mutants were expressed in *Escherichia coli* BL21-CodonPlus (DE3)-RP cells and obtained as a heat-stable supernatant of the cell extracts [30]. The amount of drebrin was estimated by Coomassie Brilliant Blue staining of gels using bovine serum albumin as the standard.

### Preparation of Actin Gels from Mouse Brain

Actin gels were prepared from mouse brains according to the method described previously by Taguchi *et al*. [31]. Briefly, mouse brains were homogenized in gelation buffer (40 mM Tris-HCl, pH 7.5, 0.5 mM ATP, 1 mM EDTA, 10 mM 2-mercaptoethanol, 0.5% Triton-X 100) by a teflon-pestle glass homogenizer and centrifuged at 100,000 g for 90 min at 4°C. The supernatant was incubated at 25°C for 60 min. After centrifuged at 10,000 g for 10 min at 25°C, the pellet was collected and resuspended with gelation buffer and then centrifuged at 20,000 g for 20 min at 4°C. The pellet was collected after centrifugation at 20,000 g for 20 min at 4°C and resuspended with MOPS buffer (10 mM MOPS, pH 6.8, 1 mM MgCl_2_, 0.1 mM EDTA, 0.1 mM EGTA, 0.5% Nonidet P-40 (NP-40). The actin gel fraction was collected and used for *in vitro* phosphorylation.

### Mass Spectrometric Analysis

Protein bands were stained using the ProteoSilver Plus Silver Stain Kit (Sigma, St. Louis, MO) according to the manufacturer’s protocol and excised from the polyacrylamide gel. After washing, the gels were digested by incubation in buffer with trypsin [32]. The tryptic digests were analyzed by an LC-MS/MS system as described previously [33]. Database search was performed using MASCOT software (version 2.2.1., Matrix Science Ltd., London) and the NCBI Refseq sequence database under the parameters as described previously [34].

### *In vitro* Phosphorylation of Drebrin

Cdk5-p35 was expressed and purified from Sf9 cells (Clontech, Palo Alto, CA) infected by Baculovirus encoding Cdk5 and p35, as described previously [35]. Drebrin at 50 αg/mL was phosphorylated by Cdk5-p35 at 37°C for 1 h in the presence of 0.1 mM [γ-^32^P]ATP. Phosphorylation was detected by autoradiography after 10% polyacrylamide gel SDS-PAGE, and the extent of phosphorylation was quantified using a FLA7000 bioimage analyzer (GE Healthcare, Tokyo, Japan).

### Cell Culture and Transfection

COS-7 and Neuro2A cells were obtained from Japanese Collection of Research Bioresources (Osaka, Japan) and maintained in Dulbecco’s modified Eagle’s medium (DMEM) containing 10% fetal bovine serum, 100 U/mL penicillin, and 0.1 mg/mL streptomycin. Cells were plated at a density of 3×10^4^ cells/cm^2^ for immunoblotting or at a density of 1.5×10^4^ cells/cm^2^ on coverslips for immunofluorescent staining. Cells were transfected with the indicated plasmids using PolyFect Transfection Reagent (Qiagen, Hilden, Germany) or Lipofectamine 2000 (Invitrogen, Carlsbad, CA) according to the manufacturer’s instructions.

### Primary Neuron Cultures, Transfection, and *in utero* Electroporation

ICR mice were purchased from Sankyo Labo. Mice were housed in a temperature-controlled room under a 12-h light/12-h dark cycle with free access to food and water. Brains were dissected from embryos at E14–16. Cortical and hippocampal neurons were prepared as described previously [36] with some modifications. Neurons were plated on polyethyleneimine-coated dishes at density of 1.25–2.5×10^5^ cells/cm^2^ for immunoblotting or 1.3–7.8×10^4^ cells/cm^2^ for immunofluorescence staining in DMEM and HAM F-12 (1∶1) supplemented with 5% fetal bovine serum and 5% horse serum. At 4 h after plating, culture medium was changed to Neurobasal Medium containing B27 Supplement and 0.5 mM L-glutamine. Half of culture medium was changed every 2 days.

Plasmid vectors were introduced into neurons at 0 DIV (days *in vitro*) by electroporation using an Amaxa Nucleofector (Lonza Japan, Tokyo, Japan) and the number and length of axon and neurites was measured as described previously [37]. Plasmid vectors were also introduced into cultured neurons by a calcium phosphate method as described previously [38].

*In utero* electroporation was performed as described previously [39]. Plasmid vectors were injected into E14 embryonic mouse brains and electroporated *in utero*. Mice were sacrificed at postnatal day 2. Brains were sliced and observed using a LSM 510 Exciter.

### Immunofluorescent Staining

Neurons and COS-7 cells were fixed with 4% paraformaldehyde in PBS for 20 min, and permeabilized with PBS containing 0.2% Triton X-100 and 5% normal goat serum for 1 h. The cells were probed with primary antibodies, anti-drebrin, anti-pS142, anti-tubulin, anti-Homer2a, or anti-myc in PBS containing 0.1% Triton X-100 and 1% normal goat serum for 1 h. After incubation with secondary antibodies and rhodamine-phalloidin in PBS containing 0.1% Triton X-100 and 1% normal goat serum for 1 h, specimens were observed using a confocal laser scanning microscope LSM 510 Exciter (Carl Zeiss, Jena, Germany).

### Laemmli’s SDS-PAGE, Phos-tag SDS-PAGE, and Immunoblotting

Laemmli’s SDS-PAGE was carried out using 7.5% polyacrylamide gels. Proteins were transferred to PVDF (Millipore, Bedford, MA) membranes using a semi-dry blotting apparatus. Phos-tag SDS-PAGE was performed with 5% or 7.5% polyacrylamide gels containing 60 αM Phos-tag acrylamide and 90 αM MnCl_2_. Proteins were transferred to PVDF membranes using a submarine blotting apparatus. Immunodetection was carried out using an enhanced chemiluminescence system (ECL; GE Healthcare) or Millipore Immobilon western chemiluminescent HRP substrate (Millipore).

### Quantification and Statistical Analysis

Immunoreaction was visualized as digital images by scanning X-ray film, and band intensities were measured using Image J software. GFP images of cultured cells expressing EGFP-drebrin were acquired with Zeiss 510 Exciter or 710 confocal microscopes. Axonal length and neurite number were measured using ZEN imaging software (Zeiss). The longest process of primary neurons at 3 DIV was defined as an axon and its length was measured. Other processes longer than 20 μm were defined as neurites and their number (per neuron) was measured. Protrusions with length of 0.5 αm to ∼5 αm on dendrites of neurons at 21 DIV were defined as dendritic spines, and the length, width, and number per 30∼50 αm primary dendrite were measured. All quantitative data were represented as mean ± SEM and were subjected to two-tailed unpaired Student’s t-test for single comparison, or one-way ANOVA analysis, followed by Tukey’s or Tukey-Kramer’s post-hoc test, for the multiple comparison. Values of p<0.05 were considered to be statistically significant.

## Results

### Drebrin is a Substrate for Cdk5-p35

**Figure 1:**
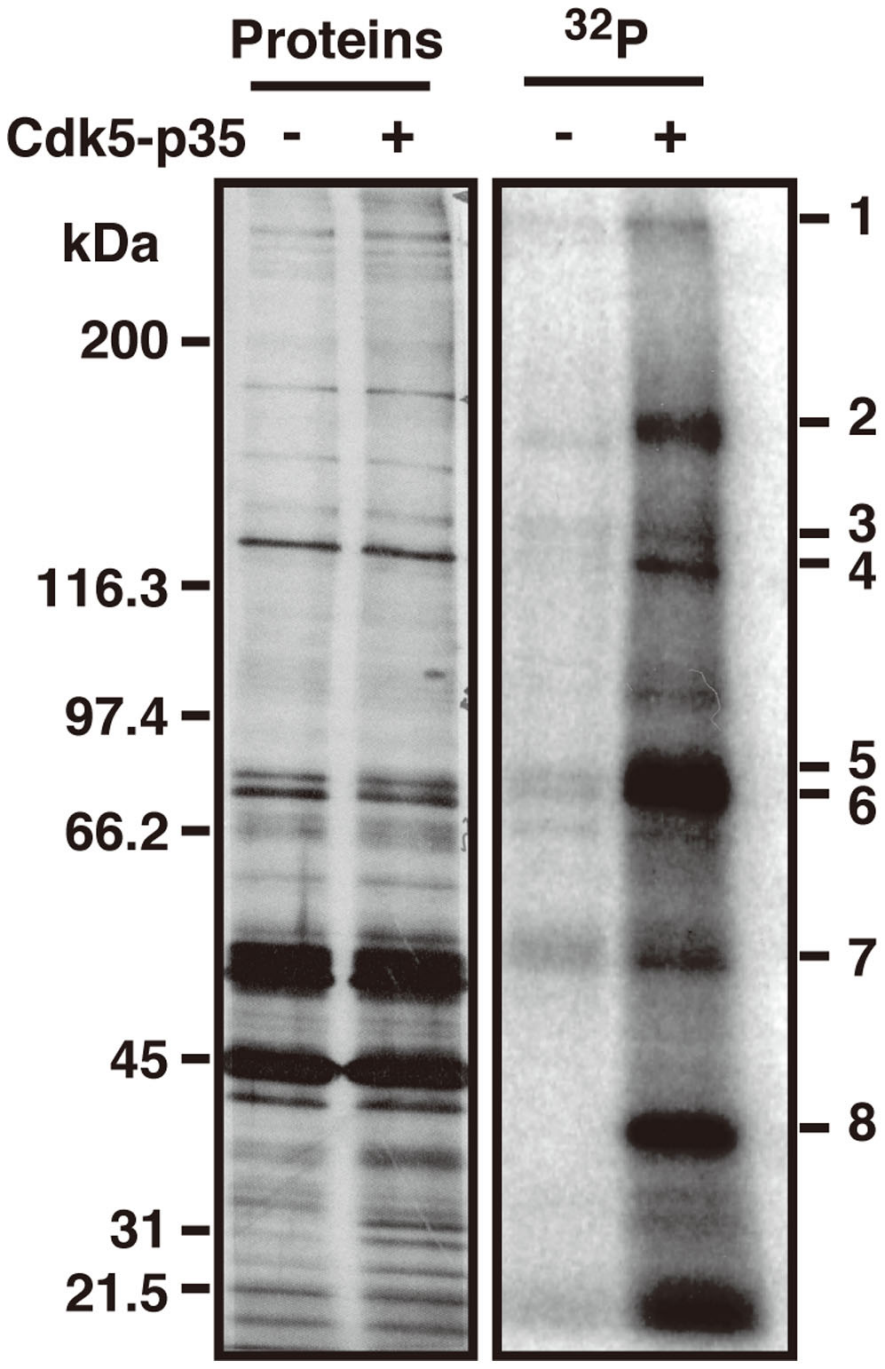
Cdk5-p35 phosphorylates Drebrin in actin gels. Actin gels were prepared from mouse brain extracts. The actin gels were incubated with Cdk5-p35 in the presence of [γ-^32^P]ATP *in vitro* at 35°C for 60 min. Protein composition is shown by silver staining of SDS-PAGE gel (left panel) and phosphorylation is shown by autoradiography (right panel). Bands 1–8 were subjected to mass spectroscopic analysis.

The actin cytoskeleton is a target of Cdk5-p35 and a number of actin-binding proteins are phosphorylated by Cdk5-p35 [12]–[14]. We hypothesized that there are more Cdk5 substrates among actin-binding proteins. We prepared actin gels from mouse brain extracts, which contain many actin binding proteins, and phosphorylated them with Cdk5-p35 *in vitro* in the presence of [γ-^32^P]ATP (Figure 1). Phosphorylated bands, which are numbered on the gel of Figure 1, were subjected to LC-MS analysis. Drebrin was identified in bands 3 and 4 as a possible candidate of Cdk5 substrates.

**Figure 2:**
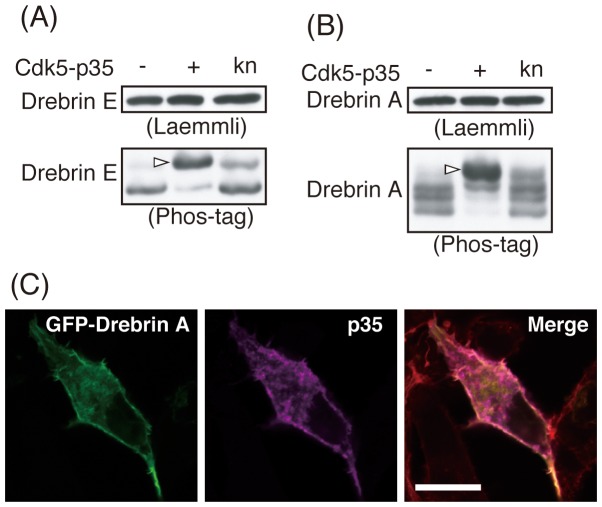
Drebrin is phosphorylated by Cdk5-p35 in cultured cells. GFP-tagged drebrin E (A) or A (B) was transfected with Cdk5-p35 or kinase-negative (kn) Cdk5-p35 in Neuro2A cells. Cells were harvested 24 h after transfection and the cell lysates were subjected to Laemmli’s (upper panels) or Phos-tag SDS-PAGE (lower panels), followed by immunoblotting with anti-GFP antibody. Expression of GFP-drebrin alone is shown in left lane (−). Arrowhead indicates drebrin band shifted upward by coexpression with Cdk5-p35. (C) GFP-drebrin A and p35 were co-expressed in COS-7 cells. At 24 h after transfection, COS-7 cells were stained with anti-myc antibody for p35. Drebrin A was detected by tagged GFP. Cells were also stained with TRITC-phalloidin for actin filaments (not shown). Merge indicates colocalization of drebrin A and p35. Scale bars, 20 μm.

Cdk5-dependent phosphorylation of drebrin was confirmed by expression in Neuro2A cells. Phosphorylation was detected with Phos-tag SDS-PAGE [40], [41], in which phosphorylated proteins show slower mobility. Both drebrin E and A appeared as a single band in Laemmli’s SDS-PAGE whether or not they were co-expressed with Cdk5-p35 (Figure 2A and B, upper panels). Drebrin E was a single band in Phos-tag SDS-PAGE when it was expressed alone in COS-7 cells, but shifted upward when co-expressed with Cdk5-p35 (Figure 2A, white arrow), indicating that drebrin E is phosphorylated by Cdk5-p35.

Drebrin A was separated into multiple bands, even in the absence of Cdk5-p35, suggesting phosphorylation of a part of drebrin in cells (Figure 2B). Co-expression with Cdk5-p35, but not kinase negative (kn) Cdk5-p35, induced a shift in the drebrin A bands to bands migrating at a higher molecular weight (Figure 2B, white arrow). These results indicate that drebrin E and A are phosphorylated by Cdk5-p35 under cellular conditions.

To confirm the interaction of drebrin with Cdk5-p35, we co-expressed GFP-drebrin A with p35 Cdk5 activator, a determinant of cellular localization of the active Cdk5-p35 complex, in COS-7 cells. p35 showed colocalization with drebrin A (Figure 2C), suggesting that drebrin interacts with Cdk5-p35.

### Ser142 and Ser342 are Cdk5-phosphorylation Sites in Drebrin

**Figure 3:**
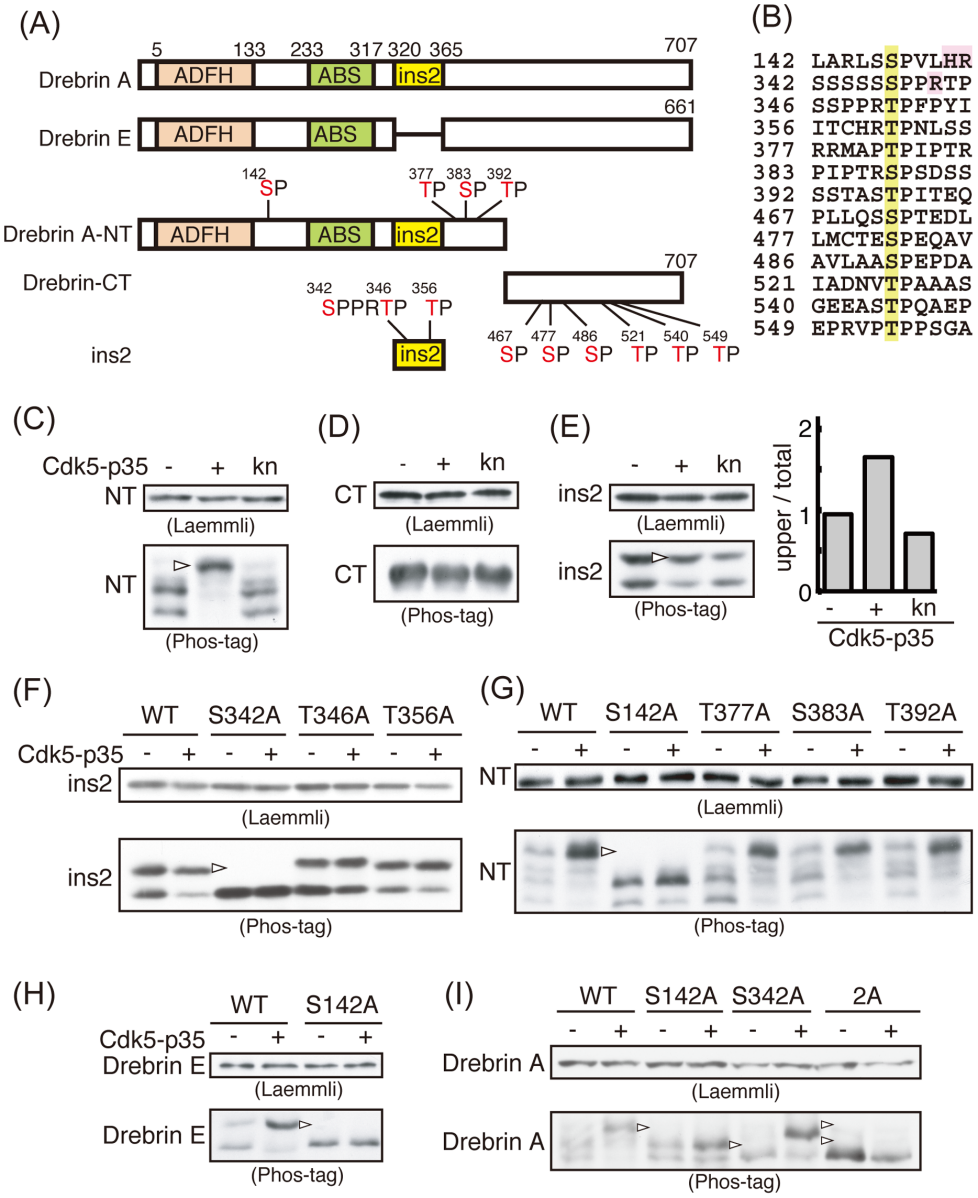
Cdk5-p35 phosphorylates drebrin at Ser142 and Ser342. (A) Schematic representation of drebrin molecules. There are two isoforms, drebrin A and drebrin E, with a difference in an insertion (ins2) of 46 amino acids in the middle region of drebrin A. Actin-depolymerizing Factor Homology domain (ADFH) is in the N-terminal region and actin binding site (ABS) is in the middle region. There are 13 and 10 (S/T)P Cdk5 consensus phosphorylation sites in drebrin A and drebrin E, respectively. Drebrin A-NT is the N-terminal fragment of drebrin A, composed of amino acids 1∼430 with 7 (S/T)P sites. Drebrin-CT is the C-terminal fragment composed of amino acids 431∼707 with 6 (S/T)P sites. The ins2 insertion contains 3 (S/T)P sites. (B) Amino acid sequences around SP or TP Cdk5 consensus sequence in drebrin. Ser or Thr in (S/T)P site is marked by yellow and basic amino acids present at two three amino acids C-terminal region are indicated by magenta. (C)–(E) Immunoblots of drebrin A-NT (C, NT), drebrin-CT (D, CT), and ins2 (E, ins2) expressed in Neuro2A cells with Cdk5-p35 or kinase negative (kn) Cdk5-p35 after Laemmli’s SDS-PAGE (upper panel) or Phos-tag SDS-PAGE (lower panel). Left lane (−) is expression of drebrin fragments in the absence of Cdk5-p35. Arrowhead indicates Cdk5-dependent phosphorylation bands. The ratio of the upper band to total bands of ins2 is shown in the right panel of (E). (F)–(I) Phosphorylation of Ala mutants at the indicated (S/T)P site on GFP-ins2 (F), GFP-drebrin A-NT (G), GFP-drebrin E (H), and GFP-drebrin A (I). Arrowheads indicate the position of phosphorylated bands, which were absent in mutants.

Cdk5-p35 is a proline-directed protein kinase [5]–[7]. Drebrin E and A have 10 and 13 (S/T)P Cdk5 consensus sequences, respectively (Figure 3A). In order to identify phosphorylation sites, we divided drebrin A into two fragments, the N-terminal fragment (drebrin A-NT) and C-terminal fragment (drebrin-CT; common to drebrin E and A), each of which has 6 or 7 (S/T)P sites (Figure 3A). We also prepared the drebrin A specific insertion, the ins2 fragment. These fragments were coexpressed with Cdk5-p35 in Neuro2A cells.

Drebrin A-NT appeared as two major bands (Figure 3C, Phos-tag in lower panel), and these bands were shifted up to a single band in the presence of Cdk5-p35. In contrast, drebrin-CT did not show any upward shift when co-expressed with Cdk5-p35 (Figure 3D; Phos-tag), indicating that there is no Cdk5 phosphorylation site in drebrin-CT. Ins2 was split into two bands, even when expressed alone, and the upper band became stronger when co-expressed with Cdk5-p35 (Figure 3E; Phos-tag). To confirm the shift, we measured the ratio of the upper band to total ins2 (upper and lower bands). Coexpression of Cdk5-p35, but not knCdk5, increased the ratio of the upper band 1.7-fold (Figure 3E, right panel). These results indicate that Cdk5-phosphorylation sites are present in drebrin A-NT and ins2.

To identify phosphorylation sites, we constructed Ala mutants of each (S/T)P site of drebrin A-NT and ins2 and expressed them in Neuro2A cells with or without Cdk5-p35. There are three (S/T)P sites in ins2 with an SPPR sequence as a preferred consensus for Cdk5 (Figure 3A). Their alanine mutants were co-expressed with Cdk5-p35 in Neuro2A cells. The shifted band of ins2, a part of drebrin A-NT, disappeared with the S342A mutant, but not with T346A and T356A, which demonstrates that Ser342 is the Cdk5 phosphorylation site (Figure 3F). Ser342 is a preferred consensus sequence for Cdk5 with Arg at two amino acids downstream from the SP sequence (Figure 3B). Then, we mutated other (S/T)P sequences (Ser142, Thr377, Ser383, and Thr392) in drebrin A-NT other than those present in ins2. The large upward shift of drebrin-NT induced by co-expression with Cdk5-p35 was lost with the S142A mutant, but not with other Ala mutants, such as T377A, S382A, or T392A (Figure 3G), indicating that Ser142 is a Cdk5 phosphorylation site. This site also has His and Arg basic amino acids at the three and four C-terminal side of the SP sequence (Figure 3B). The S142A mutant shifted upward whether Cdk5-p35 was coexpressed or not, but this shift might be due to phosphorylation at Ser342 in the drebrin-NT.

To confirm the phosphorylation of these sites in full-length drebrin, we mutated Ser142 in drebrin E and Ser142 and Ser342 in drebrin A to Ala (Figure 3G, H). Introduction of the S142A mutation into drebrin E caused the shift to disappear even when co-expressed with Cdk5-p35 (Figure 3H). In the case of drebrin A, single mutation at Ser142 reduced the shift considerably, and another single Ala mutation at Ser342 decreased the shift moderately (Figure 3I). The Cdk5-dependent band shift was completely lost with the double mutant of drebrin A (Figure 2A, 3H). The sum of both corresponded to the upward shift of drebrin A-WT, indicating that these two sites cover most of Cdk5 phosphorylation sites. Taken together, these results show clearly that drebrin A is phosphorylated at Ser142 and Ser342, and that drebrin E is phosphorylated at Ser142 by Cdk5-p35.

### *In vitro* Phosphorylation of Drebrin by Cdk5-p35

**Figure 4:**
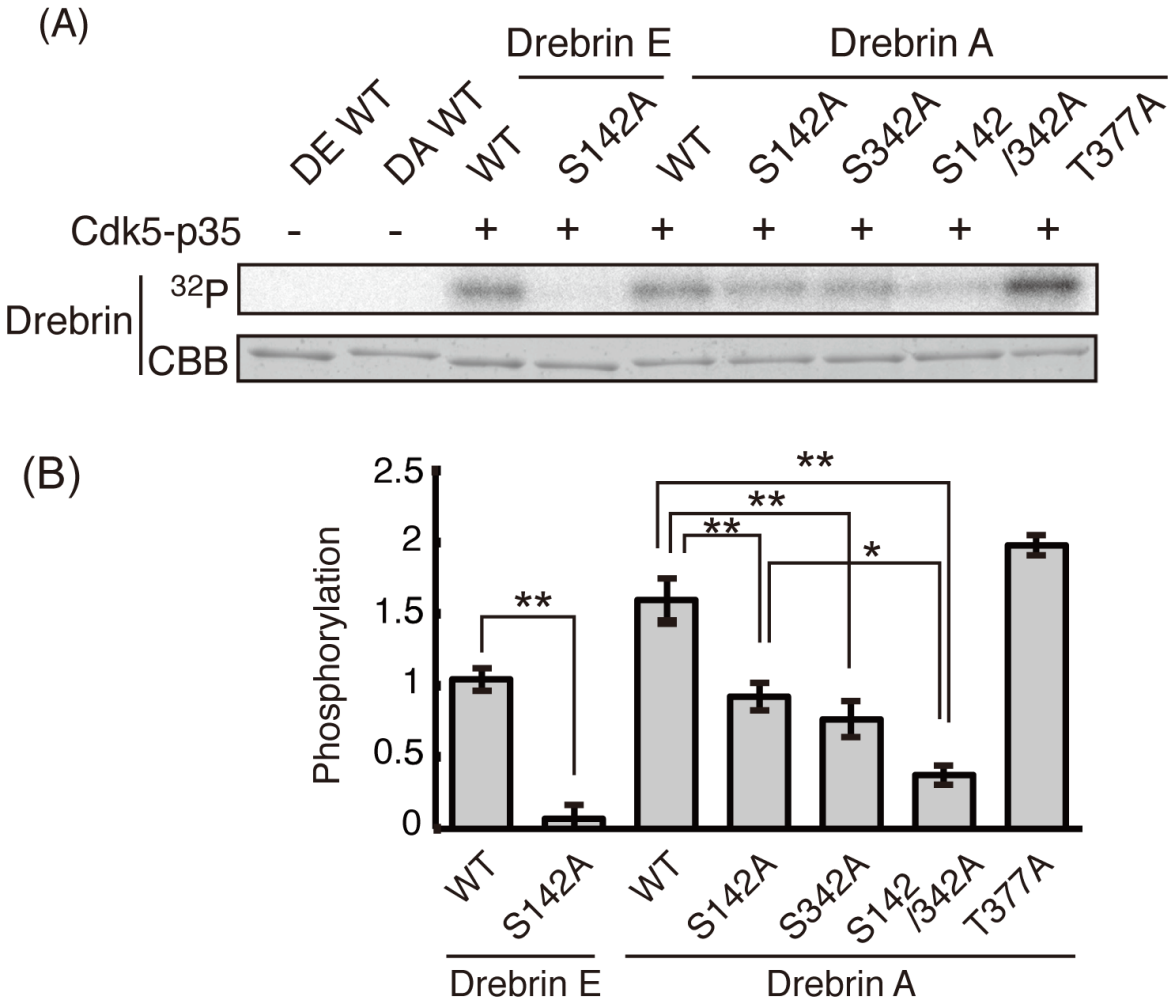
*In vitro* phosphorylation of drebrin at Ser142 and Ser342 by Cdk5-p35. (A) Drebrin E, A, or their Ala mutants was incubated with Cdk5-p35 at 37°C for 1 h in the presence of [γ-^32^P]ATP. Phosphorylation was detected by autoradiograph (^32^P-drebrin) after SDS-PAGE. (B) Quantitation of phosphorylation. Phosphate incorporation into respective drebrin molecule was estimated using a FLA7000 bioimage analyzer and expressed as the ratio to drebrin WT after normalization with drebrin protein measured by Coomassie blue staining. (n = 3, ns, not significant, * *P*<0.05, ** *P*<0.01, student’s *t*-test for Drebrin E and Dunnet’s test for Drebrin A).

A part of drebrin shifted upward without co-expression of Cdk5-p35 (Figure 3), suggesting the possibility that drebrin is phosphorylated by other protein kinases which are stimulated by overexpression of Cdk5-p35. To exclude this possibility, we performed *in vitro* phosphorylation by incubating recombinant drebrin with purified Cdk5-p35 in the presence of [α-^32^P]ATP. Drebrin E-WT was phosphorylated by Cdk5-p35 and its phosphorylation was completely abolished with the S142A mutant (Figure 4A, B). When drebrin A-WT was phosphorylated by Cdk5-p35 and S142A or S342A, but not T377A, the mutation decreased phosphorylation and the S142/342A double mutation further decreased phosphorylation. Quantitative measurement indicated that Ser142 is a single Cdk5 phosphorylation site in drebrin E, Ser142, and Ser342, which are additively phosphorylated in drebrin A, which constitutes most Cdk5 phosphorylation sites in drebrin A.

### Cdk5 Phosphorylates Drebrin in Neurons

**Figure 5:**
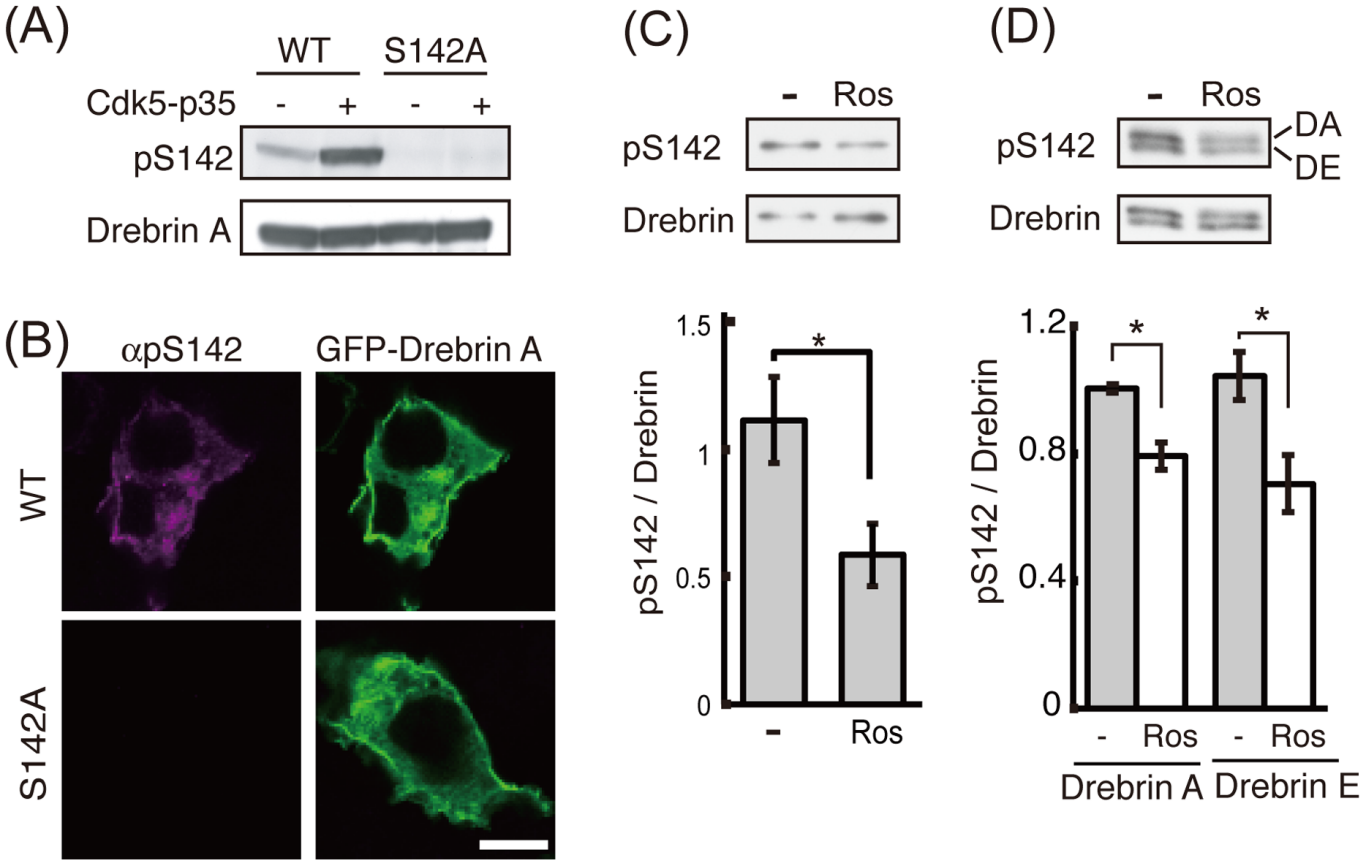
Cdk5-dependent phosphorylation of drebrin at Ser142 in neurons. (A) The specificity of anti-phospho-Ser142 (pS142). Drebrin A-WT or -S142A was expressed with or without Cdk5-p35 in Neuro2A cells. The cell lysates were blotted with anti-pS142 (pS142) or anti-GFP (drebrin). (B) GFP-drebrin was transfected in Neuro2A cells and cells were stained with anti-pS142. Drebrin was visualized with GFP. Scale bar, 20 μm. (C) and (D) Phosphorylation of drebrin at Ser142 in neurons. Primary cortical neurons at 2 DIV were treated with 20 αM Cdk5 inhibitor roscovitine (Ros) for 24 h. The cell lysates were immunoblotted with anti-pS142 or anti-drebrin (upper) and quantified (lower) (n = 4, * *P*<0.05, student’s *t*-test). (D) Primary cortical neurons at 14 DIV were treated with roscovitine for 12 h. Immunoblottings are shown in the upper panel and quantification is shown in the lower panel (n = 3, * *P*<0.05, student’s *t*-test).

To confirm *in vivo* phosphorylation of drebrin, we produced anti-phospho-drebrin antibody at the Ser142 site. Anti-pS142 antibody reacted with drebrin A WT but not drebrin A S142A (which were expressed in Neuro2A cells with Cdk5-p35) by immunoblotting (Figure 5A) and immunostaining (Figure 5B), indicating the specificity of the antibody.

We further examined phosphorylation of drebrin in primary cortical neurons at 2 DIV for drebrin E (Figure 5C) and 14 DIV for drebrin E and A (Figure 5D). Anti-pS142 antibody reacted with both endogenous drebrin E and drebrin A. These results indicate that both drebrins are phosphorylated at Ser142 in neurons. To determine if the phosphorylation is Cdk5-dependent, we treated neurons with Cdk5 inhibitor roscovitine. Roscovitine decreased phosphorylation of drebrin E and A at Ser142 (Figure 5C, D, upper panels). Quantitative measurements confirmed the significant decrease in anti-pS142 reactivity after roscovitine treatment (Figure 5C, D, lower panels); however, anti-pS142 reactivity was not abolished completely. These results indicate that drebrin is phosphorylated at Ser142 by Cdk5 in neurons but these sites can be phosphorylated by other protein kinases.

**Figure 6:**
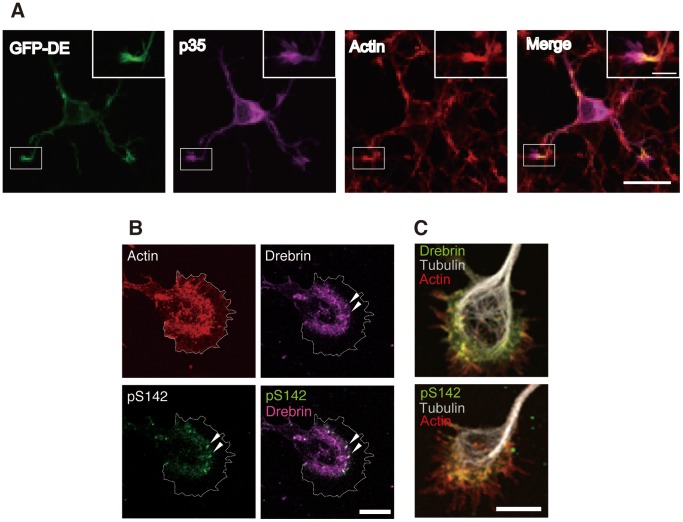
Co-localization of drebrin, p35, and actin filaments in neurons. (A) GFP-drebrin E (GFP-DE) was co-transfected with p35-myc in primary cortical neurons at 1 DIV and their cellular distribution was examined at 3 DIV. Actin filaments were stained with TRITC-phalloidin and p35 was labeled with anti-myc antibody. Higher magnification of a neurite (square) is shown in insets. Scale bars, 20 μm and 5 μm (inset). (B) Mouse hippocampal neurons in culture at 3 DIV were immunostained with anti-pS142 and anti-drebrin antibodies. Actin filaments were stained with TRITC-phalloidin. Arrowheads indicate dotted stainings with anti-pS142. The growth cone area is indicated by the white line. Scale bar, 10 μm. (C) Co-immunostaining of growth cone of hippocampal neurons with anti-tubulin (white), anti-drebrin (green in upper), or anti-pS142 (green in lower), and actin filaments with TRITC-phalloidin (red). Bar, 10 αm.

To investigate the interaction of drebrin with Cdk5-p35 in neurons, GFP-drebrin E was transfected in neurons with the Cdk5 activator p35 at 2 DIV and their co-localization was observed at 3 DIV. GFP-drebrin E was found in the shaft of neurites and in the growth cone. The distribution of GFP-drebrin E overlapped mostly with actin filaments in neurons (Figure 6A) and p35 showed predominant localization at perinuclear regions in the cell body as well as at the tip of neurites (Figure 6A), as was shown previously [42], [43]. Higher magnification of the growth cone area is shown in insets of Figure 6. Drebrin was found at a part of actin filaments and co-localized partly with p35 in growth cone, suggesting a possible interaction of drebrin with Cdk5-p35 in neurons.

Additionally, we studied the localization of phosphorylated endogenous drebrin in the growth cone of cultured hippocampal neurons and found that drebrin was not detected at the peripheral region; however, drebrin was found in the transition zone where actin filaments were highly enriched (Figure 6B). Interestingly, phospho-drebrin was localized at the distal part of total drebrin in the growth cone. Co-staining with tubulin is shown in Figure 6C. In this fan-like growth cone, microtubules were bent and curled at the region where drebrin was present. Phospho-drebrin was also found in the center of growth cone where microtubules appeared to terminate.

### Phosphorylation of Drebrin does not Affect Neurite and Spine Formation

**Figure 7:**
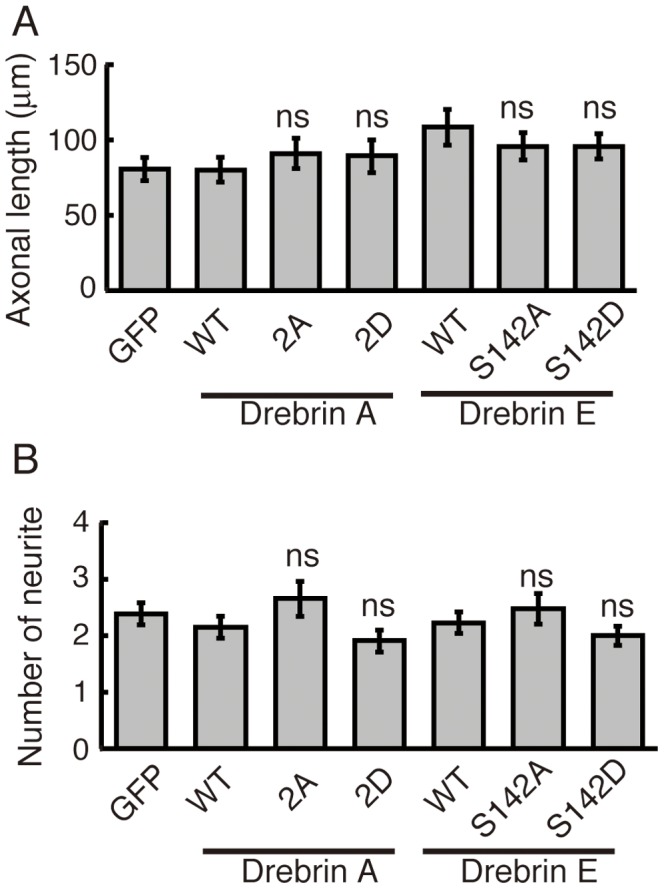
Mutation at Cdk5-phosphorylation sites does not affect the neurite growth by drebrin E. GFP-drebrin E-WT, -S142A, or -S142D as well as GFP-drebrin A-WT, -2A (S142/342A), or -2D (S142/342D) was co-transfected with DsRed in primary cortical neurons at 0 DIV, and axonal length (A) and the number of neurites (B) of GFP-expressing neurons were measured at 2 DIV (31 neurons for GFP, 39 neurons for drebrin E-WT, 40 neurons for drebrin E-S142A, 37 neurons for drebrin E-S142D, 20 neurons for drebrin A-WT, 20 neurons for drebrin A-WT, 20 neurons for drebrin A-2A, 21 neurons for drebrin A-2D, ns, not significant, Tukey-Kramer’s test).

The finding of phospho-drebrin localization in the growth cone prompted us to study the effect of phosphorylation on neurite outgrowth. We examined the role of drebrin phosphorylation on neurite formation by expressing GFP-drebrin E-WT, non-phosphorylation mutant GFP-drebrin E-S142A, and phospho-mimic mutant GFP-drebrin E-S142D in cultured cortical neurons. Drebrin constructs were transfected in primary neurons at 0 DIV and axonal length and neurite number were measured at 2 DIV. However, we did not observe differences in axonal length between WT and mutants, S142A and S142D (Figure 7A). Even if drebrin A-WT and its 2A or 2D mutant was used, no significant difference was detected between them (Figure 7A). We also measured the number of neurites in neurons expressing drebrin mutants (Figure 7B). Although the number of neurites appeared to increase slightly with Ala mutants, no significant statistical difference was found.

**Figure 8:**
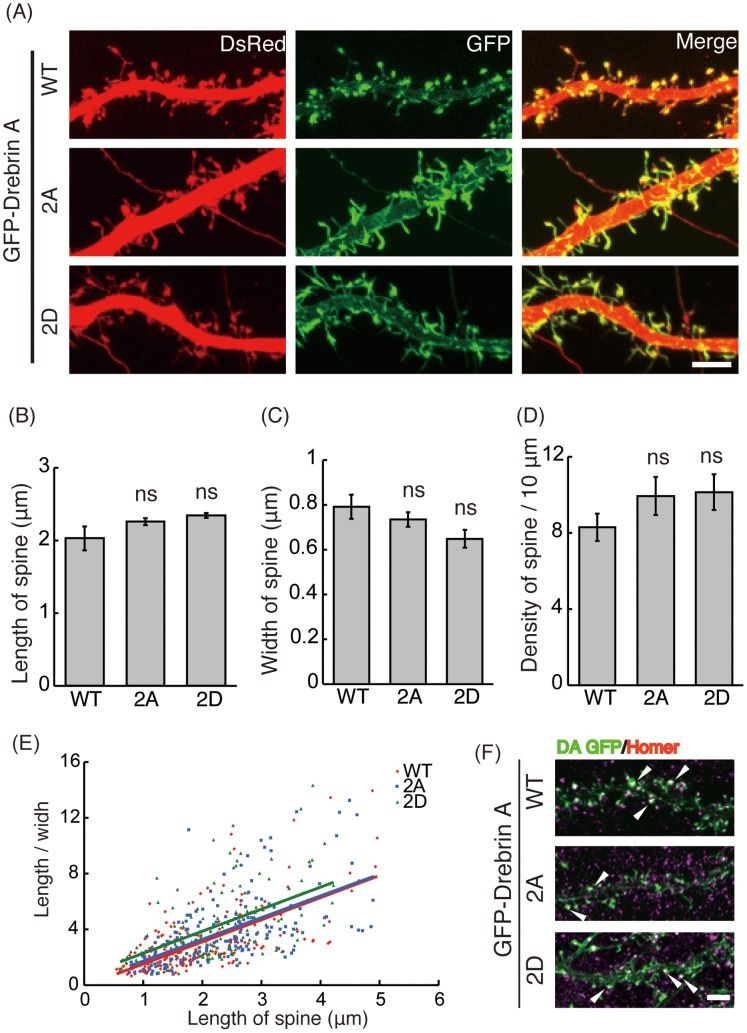
Effect of Cdk5-phosphorylation of drebrin in spine formation. (A) GFP-drebrin A-WT, -2A (S142/342A) or -2D (S142/342D) was co-transfected with DsRed in primary hippocampus neurons at 9 DIV, and spines on primary dendrites were examined at 21 DIV. Scale bar, 5 μm. (B)-(D) Length (B), width (C), and density (D) of dendritic spines in GFP-positive neurons were measured (n = 194 spines from 6 neurons for drebrin A-WT, 183 spines from 5 neurons for drebrin A-2A, 120 spines from 4 neurons for drebrin A-2D, ns, not significant, Tukey-Kramer’s test). (E) The length and width relationship of spines. The ratio of length to width was plotted against the length of spines, which were counted in (B)–(D). Lines of length-width relationship were obtained by the least square regression calculation. (F) Co-localization of drebrin with Homer2a. GFP-drebrin A-WT, -2A or -2D was transfected in primary hippocampus neurons at 9 DIV, and their localization with Homer2a in spines was examined at 21 DIV. Homer2a was stained with anti-Homer2a [50]. Arrowheads indicate co-localization of drebrin A and Homer2a. Scale bar, 5 αm.

Next, we evaluated dendritic spine morphology. We expressed GFP-drebrin A-WT and its 2A and 2D mutants in cultured hippocampal neurons at 9 DIV. DsRed was co-expressed in order to identify the shape of spines on dendrites of transfected neurons (Figure 8A). The length, width, and number of spines were measured at 21 DIV (Figure 8B–D). Since expression of drebrin A-WT changed spine structure as described previously [17], we estimated the effect of phosphorylation by comparing the difference between drebrin A-WT and mutants, 2D and 2A. Although we suspected that longer protrusions were formed in neurons expressing mutant drebrin E, quantification indicated that length, width, and density were not significantly altered by 2A or 2D mutation (Figure 8B–D). We confirmed this finding in the length and width relationship plot (Figure 8E). Spines took similar structures when either drebrin E-WT, -2A, or -2D were overexpressed.

Drebrin is recruited to spines by Homer2a, a scaffold protein in spines. We examined the effect of drebrin colocalization with Homer2a by transfection of drebrin A-WT, -2A, or- 2D in hippocampal neurons. Drebrin A-WT co-localized with Homer2a in spines as previously reported [26]. Drebrin A-2A and -2D also showed co-localization with Homer2a (Figure 8F); we did not find a difference in colocalization between drebrin A-2A and -2D.

### Drebrin Mutants at Cdk5-phosphorylation Sites Suppresses Radial Migration of Cortical Neurons in Embryo

**Figure 9:**
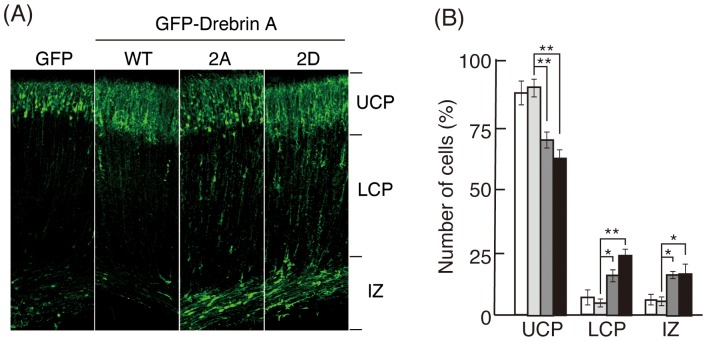
*In utero* migration of cortical neurons expressing drebrin WT, 2A, or 2D. (A) Plasmids encoding GFP, GFP-drebrin A-WT, -2A, or -2D were introduced into embryonic cortical neurons at E14 by *in utero* electroporation. Mice were sacrificed at P2, and positions of neurons were visualized by GFP. (B) The number of EGFP-expressing neurons in upper cortical plate (UCP), lower cortical plate (LCP), and intermediate zone (IZ; right side of A), were measured and expressed as the % ratio of total number of GFP-labeled neurons {n = 5 sections for GFP (white), 7 sections for drebrin A-WT (light gray), 5 sections for drebrin A-2A (dark gray), 6 sections for drebrin A-2D (black), * *P*<0.05, ** *P*<0.01, Tukey’s test}.

Drebrin has been shown to be involved in neuronal migration (Dun et al., 2012; K.N. personal communication); therefore, we examined the effect of phosphorylation on the migration of embryonic cortical neurons using *in utero* electroporation. GFP-drebrin A-WT or -2A or -2D mutant was introduced into neuronal progenitors in the ventricular zone at embryonic day 14 (E14). Their migration to cortical plate was examined at P2. Most of neurons expressing GFP or GFP-drebrin WT migrated to upper cortical plate (Figure 9A, left two panels), whereas a proportion of neurons expressing drebrin A-2A or -2D mutant were observed at the intermediate zone (Figure 9A, right two panels).

For quantification, we divided the cerebral cortex into three zones: upper cortical plate (UCP), lower cortical plate (LCP), and intermediate zone (IZ), as shown in the right side of Figure 9A. Most neurons expressing GFP or drebrin WT migrated up to UCP (Figure 9B, GFP and WT). The ratio of both drebrin A-2A and drebrin A-2D was decreased in UCP with a concomitant increase of drebrin A-2D and drebrin A-2A in LCP and IZ (Figure 9B). These results suggest that reversible phosphorylation and dephosphorylation of drebrin is required for the migration of cortical neurons in embryonic brains.

## Discussion

Here, we demonstrated that the phosphorylation of drebrin occurs at Ser142 and Ser342 by Cdk5, and also described the roles of Ser142 and Ser342 in neuronal migration. We first identified drebrin as a protein in actin gels phosphorylated by Cdk5 by mass spectrometric analysis, and then further determined Ser142 as a phosphorylation site common in drebrin E and A, and Ser342 as a drebrin A-specific site by Phos-tag analysis in combination with Ala mutants. Phosphorylation was confirmed in neurons and detected in a subset of drebrin present at the distal region of total drebrin in the transition zone of the growth cone, which is involved in migration of embryonic cortical neurons. These results indicate that Cdk5 regulates neuronal migration through phosphorylation of drebrin in growth cone of processes in migrating neurons.

### *In vitro* and *in vivo* Phosphorylation of Drebrin at Ser142 and Ser342

Phosphorylation of drebrin has previously been described [23]–[25], but the exact sites, kinase, and role had not yet been determined. We identified drebrin among Cdk5-phosphorylated proteins in actin gels prepared from mouse brain extracts, and identified Ser142 and Ser342 as phosphorylation sites. Ser142 is located at the C-terminal site of ADF domain and Ser342 is in the insertion sequence found only in drebrin A. These sites were indeed phosphorylated by Cdk5 in neurons, but Cdk5 was not the only kinase phosphorylating them; phosphorylation of these amino acids was suggested in parietal cells of gastric mucosa by MALDI analysis [23]. It has been previously suggested that Cdk5 activity is specific to post-mitotic neurons, but the extra-neuronal activity of Cdk5 has recently been demonstrated [44]. It may be interesting to study the expression of Cdk5 and its activators in parietal cells to obtain further information on the drebrin kinase.

During the preparation of this manuscript, phosphorylation of drebrin at Ser142 by Cdk5 was reported [45]. Their mass spectrometry results list several other possible phosphorylation sites (supplemental Table 1 of Worth *et al*., [45]). Thr331 and Ser337 in human drebrin E [45], which correspond to Thr377 and Ser383 of rat drebrin A respectively (this study), are in the (Ser/Thr)-Pro sequence, and their phosphorylation has been examined here using their Ala mutants. Although they were not major Cdk5 phosphorylation sites, they were in fact phosphorylated in COS-7 cells as demonstrated by the reduced mobility shift of Ala mutants. Taken together, these results indicate that drebrin is phosphorylated at several (Ser/Thr)-Pro sequences, and, among them, Ser142 and Ser324 are targeted by the Cdk5 activity in neurons.

### A Role of Drebrin Phosphorylation in Neurite Formation

Phosphorylation at Ser142 was detected in a fraction of drebrin in the growth cone. The growth cone is the motile structure at the tip of neurites which lead the extension and navigate the direction, and is divided into three parts: the central domain, which includes microtubules elongating from neurite shaft, the peripheral domain, composed primarily of filopodia and lamellipodia, in which actin filaments are organized in parallel bundles and a mesh-like network, respectively, and the transitional domain at the interface of peripheral and central domains where actin filaments accumulate. Depending on the outgrowth stage, the growth cone shows two different images, the filopodial growth cone or the lamellipodial growth cone [3]. Under our culture conditions, cortical neurons showed mostly lamellipodial growth cone with few filopodia. Although drebrin is a side-binding protein of actin filaments [15], [16], the distribution of drebrin is not completely identical to that of actin filaments. Our finding that the localization of drebrin is at the transition zone is consistent with previous studies [19], [20]. Interestingly, phosphorylated drebrin was found at the distal part of total drebrin in the transition zone.

A similar localization of phosphorylated drebrin at Ser142 was shown by Worth *et al*. [45]. They demonstrated that drebrin is phosphorylated at the base of filopodia, whereas our results showed that drebrin is phosphorylated in the absence of distinct filopodial actin bundles. In our cultured neurons, phospho-drebrin showed a dotted distribution, which is similar to puncta of phospho-drebrin in stage I neurons prior to neurite extension [45]. Filopodial actin bundles may then recruit phosphorylated drebrin to elongate distribution along them in stage II or III neurite-extending neurons. In either case, only part of drebrin is phosphorylated at Ser142 in the growth cone, raising the question of how phosphorylation is regulated.

p35 binds to and activates Cdk5 but also determines the cellular localization of the active Cdk5 complex [28]. p35, whose localization represents the active Cdk5, was seen at the region ahead of the microtubule–rich central region of the growth cone, indicating the proximity of drebrin to the active Cdk5. These results suggest that it is likely that Cdk5-p35 is the protein kinase phosphorylating drebrin in the growth cone. It may be worth noting that the Cdk5 activity in the growth cone is regulated by Sema3A signaling which is involved in axonal guidance [46].

Overexpression or knockdown of drebrin has been shown to suppress and stimulate neurite outgrowth, respectively [20]. The localization of phospho-drebrin in the growth cone prompted us to study the effect of phosphorylation on neurite outgrowth or neurite number; however, we could not detect the effect of 2A or 2D mutant on neurite outgrowth. In contrast, Worth *et al.* [45] showed that S142A mutant drebrin hinders and S142D drebrin enhances neuritogenesis, compared to wild type drebrin. This difference may be due to the types of experimental conditions in each study. While they examined the effect of drebrin mutants at Ser142 in neuritogenesis using the replating assay, we observed neurite length in cultured neurons after transfection. Moreover, our culture conditions, in which growth cones had few filopodia, might obscure the effect of drebrin phosphorylation in neurite outgrowth.

### Function of Drebrin Phosphorylation in Neuronal Migration

It was recently reported that drebrin is required for the correct migration of oculomotor neurons in chick embryonic brains by growth cone formation and navigation [21]. Further knockdown of drebrin by *in utero* electroporation of shRNA inhibited the migration of cortical neurons (K.N. *et al*., personal communication). Based on these data, we examined the effect of drebrin phosphorylation on neuronal migration. We introduced 2A or 2D mutant or WT drebrin A into differentiating neurons at the ventricular zone of the embryonic mouse brain. From the distribution pattern of phospho-drebrin in the growth cone, we initially hypothesized that phospho-mimic drebrin would inhibit migration; however, drebrin A-2A, as well as -2D, decreased migration compared to drebrin A-WT, whose overexpression did not affect the migration. These results suggest the reversible phosphorylation and dephosphorylation of drebrin is important for neuronal migration.

Mice lacking Cdk5 or p35 have inverted neuronal layers in the neocortex [8], [9], which is caused by migration deficiency of newborn neurons; later born neurons cannot migrate past earlier migrated neurons and settle below them, resulting in an outside-in order of layering from the inside-out layers of the WT mouse brain. Neuronal migration consists of, at least, three types of processes: multipolar migration, locomotive movement, and somal translocation [1], [2], [47]. It has been suggested that Cdk5 regulates the migration at several processes through dynamic reorganization of cytoskeletons [1].

Cdk5 is involved in multipolar–bipolar transition [48]. Xie *et al*. [49] recently reported that growth cone activity is important for multiple-bipolar transition of migrating neurons to search for glial scaffold and that this activity is regulated by Cdk5 via WAVE2 phosphorylation. Nevertheless, in this paper, the authors noted that WAVE2 was not the sole substrate for Cdk5 from a more severe impairment of Cdk5-knockdown or overexpression of the dominant negative form, compared with the overexpression of nonphosphorylation form of WAVE2 (Xie *et al.*, 2013). Drebrin could be another target of Cdk5 in the growth cone of neurons at the time as the multipolar-bipolar transition. The results that neurons expressing drebrin A-2A or -2D showed impairment in neuronal migration support this hypothesis.

Cdk5 has previously been described as a direct regulator of the microtubule cytoskeleton in radial neuronal migration [1]. As described above, however, the regulation of actin filaments by Cdk5 has also been shown to be important [49]. To perform the complicated processes involved in migratory movements, neurons have to regulate both cytoskeletons in a coordinated fashion. Drebrin, originally found to be an actin-binding protein, has been shown to interact with microtubule plus end binding protein EB3 to modulate the invasion of microtubules into the growth cone [19]. Thus, drebrin may be a protein component to mediate the interaction between microtubules and actin filaments. Cdk5 would regulate dynamic remodeling of microtubules and actin filaments coordinately through phosphorylation of drebrin.
